# Supplementary material for: Rapid diversification associated with a macroevolutionary pulse of developmental plasticity
Source: eLife. 2015 Feb 4;4:e05463. doi: 10.7554/eLife.05463 (PMC4357287; doi:10.7554/eLife.05463)
Supplement: Table 1—source data 1. — DOI: http://dx.doi.org/10.7554/eLife.05463.005 [file elife05463s001.docx]

**Table 1-source data 1. Environmental induction of the Eu morph in dimorphic species.** Results for individual replicates (plates) are shown.

| Dimorphic nematode species | Treatment | | | Control | | |
| --- | --- | --- | --- | --- | --- | --- |
|  | Eu, n | St, n | Eu, % | Eu, n | St, n | Eu, % |
| Deprivation of microbial food and presence of *C. elegans* larvae (“prey” treatment) | | | | | | |
| *Allodiplogaster* sp. 1 (RS1982) | 12 | 0 | 100 | 0 | 26 | 0 |
|  | 23 | 0 | 100 | 0 | 21 | 0 |
|  | 13 | 0 | 100 | 0 | 18 | 0 |
| *Allodiplogaster sudhausi* (SB413B) | 30 | 0 | 100 | 5 | 81 | 6 |
|  | 30 | 2 | 94 | 0 | 67 | 0 |
|  | 55 | 1 | 98 | 2 | 69 | 3 |
| *Fictor stercorarius* (RS9003) | 6 | 0 | 100 | 0 | 15 | 0 |
|  | 8 | 1 | 89 | 0 | 12 | 0 |
|  | 10 | 0 | 100 | 0 | 9 | 0 |
| *Micoletzkya inedia* (RS5605) | 17 | 4 | 81 | 0 | 40 | 0 |
|  | 61 | 0 | 100 | 0 | 51 | 0 |
|  | 23 | 1 | 96 | 0 | 52 | 0 |
| *Micoletzkya japonica* (RS5524B) | 34 | 0 | 100 | 0 | 72 | 0 |
|  | 46 | 4 | 92 | 0 | 73 | 0 |
|  | 39 | 6 | 87 | 0 | 55 | 0 |
| *Mononchoides* sp. 1 (RS5441) | 55 | 3 | 95 | 5 | 59 | 8 |
|  | 43 | 0 | 100 | 1 | 36 | 3 |
|  | 38 | 0 | 100 | 9 | 43 | 17 |
| *Mononchoides* sp. 3 (RS9008) | 49 | 0 | 100 | 2 | 37 | 5 |
|  | 18 | 0 | 100 | 0 | 25 | 0 |
|  | 26 | 0 | 100 | 4 | 26 | 13 |
| *Neodiplogaster* sp. (RS9009) | 41 | 1 | 98 | 0 | 47 | 0 |
|  | 107 | 0 | 100 | 0 | 87 | 0 |
|  | 62 | 0 | 100 | 0 | 45 | 0 |
| Crowding and deprivation of food (“starved” treatment) | | | | | | |
| *Diplogasteriana* n. sp. (RS9000) | 12 | 18 | 40 | 0 | 200 | 0 |
|  | 3 | 19 | 14 | 0 | 200 | 0 |
|  | 3 | 21 | 13 | 0 | 200 | 0 |
| *Koerneria luziae* (RS5613) | 6 | 194 | 3 | 0 | 200 | 0 |
|  | 16 | 184 | 8 | 0 | 200 | 0 |
|  | 12 | 188 | 6 | 0 | 200 | 0 |
| *Parapristionchus giblindavisi* (RS5555B) | 31 | 48 | 39 | 5 | 95 | 5 |
|  | 34 | 66 | 34 | 7 | 93 | 7 |
|  | 29 | 71 | 29 | 5 | 95 | 5 |
