# Supplementary material for: Rapid diversification associated with a macroevolutionary pulse of developmental plasticity
Source: eLife. 2015 Feb 4;4:e05463. doi: 10.7554/eLife.05463 (PMC4357287; doi:10.7554/eLife.05463)
Supplement: Figure 2—source data 1. — (A) Origins of strains are coded as follows: CGC, Caenorhabditis Genetics Center; JB, Baldwin lab (U. California, Riverside); MV, Viney lab (U. Bristol); NK, Kanzaki lab; RGD, Giblin-Davis lab (U. Florida-IFAS); RS, Sommer lab; SB, Sudhaus lab (Freie Universität Berlin). For other strains, references are given. nc, not culturable. (B) GenBank accession numbers for gene sequences analyzed in this study. Sequences shorter than 200 bp (with accession numbers beginning with ‘VS’) are available at www.pristionchus.org/download/suppSusoy2014.html. DOI: http://dx.doi.org/10.7554/eLife.05463.007 [file elife05463s002.docx]

**Figure 2-source data 1a. Nematode taxa used in this study, with isolation details given.** Origins of strains are coded as follows: CGC, Caenorhabditis Genetics Center; JB, Baldwin lab (U. California, Riverside); MV, Viney lab (U. Bristol); NK, Kanzaki lab; RGD, Giblin-Davis lab (U. Florida-IFAS); RS, Sommer lab; SB, Sudhaus lab (Freie Universität Berlin). For other strains, references are given. nc, not culturable.

| Nematode species | Strain | Isolation source | Location | Origin |
| --- | --- | --- | --- | --- |
| Diplogastridae | | | | |
| *Acrostichus* cf. *nudicapitatus* | RS5083 | Soil | Breitenholz, Germany | RS |
| *Acrostichus halicti* | JB120 | *Halictus ligatus* | Davis, CA, USA | JB |
| *Acrostichus rhynchophori* | RGD193 | *Rhynchophorus cruentatus* | Florida, USA | RGD |
| *Allodiplogaster hylobii* | RS5529 | *Hylobius abietis* | Tübingen, Germany | RS |
| *Allodiplogaster* sp. 1 | RS1982 | *Melolontha melolontha* | Usedom, Germany | RS |
| *Allodiplogaster sudhausi* | SB413 | Soil | Israel | SB |
| *Butlerius* sp. | nc | Frass from passalid beetle | New Caledonia | RS |
| *Diplogasteriana* n. sp. | RS9000 | *Nosodendron unicolor* | Nebraska, USA | RS |
| *Diplogasteriana schneideri* | RS5440 | *Nosodendron fasciculare* | Stuttgart, Germany | RS |
| *Diplogasteroides magnus* | RS1983 | *Melolontha melolontha* | Tübingen, Germany | RS |
| *Diplogasteroides* sp. | RS5444 | *Holotrichia* sp. | Kobe, Japan | RS |
| *Diplogastrellus gracilis* | SB306 | - | Sweden | SB |
| *Diplogastrellus* sp. | RS5608 | Frass from *Leptaulax* sp. | Christmas Island | RS |
| *Eudiplogasterium levidentum* | nc | Cow dung | Tübingen, Germany | RS |
| *Fictor* sp. 1 | RS9001 | *Trox* sp. | Cambodia | RS |
| *Fictor* sp. 2 | RS9002 | *Trox* sp. | Arizona, USA | RS |
| *Fictor stercorarius* | RS9003 | Dung beetle | Tübingen, Germany | RS |
| *Fuchsnema halleri* | RS5531 | *Ips typographus* | Tübingen, Germany | RS |
| *Fuchsnema* sp. | RS5537 | *Gnathotrichus materiarius* | Tübingen, Germany | RS |
| *Koerneria luziae* | RS5613 | Frass from *Dorcus ritsemae* | Indonesia | RS/NK |
| *Koerneria* sp. | RS9004 | Frass from *Dorcus rectus* | Tsukuba, Japan | RS/NK |
| *Leptojacobus dorci* | RS9005 | Frass from *Dorcus ritsemae* | Indonesia | RS/NK |
| *Levipalatum texanum* | RS5280 | *Cyclocephala* sp. | Texas, USA | RS |
| *Mehdinema alii* | nc | *Gryllodes sigillatus* | Riverside, CA, USA | RS |
| *Micoletzkya buetschlii* | RS9006 | *Ips typographus* | Tübingen, Germany | RS |
| *Micoletzkya inedia* | RS5605 | *Dendroctonus ponderosae* | Arizona, USA | RS |
| *Micoletzkya japonica* | RS5524 | *Dryocoetes uniseriatus* | Kasumigaura, Japan | RS |
| *Micoletzkya* sp. | RS5562 | *Dryocoetes autographus* | Tübingen, Germany | RS |
| *Mononchoides* sp. 1 | RS5441 | *Geotrupes* sp. | Corsica, France | RS |
| *Mononchoides* sp. 2 | RS9007 | Scarabaeidae sp. | Mexico | RS |
| *Mononchoides* sp. 3 | RS9008 | - | New Caledonia | RS |
| *Neodiplogaster crenatae* | nc | *Scolytoplatypus daimio* | Shirakami, Japan | (1) |
| *Neodiplogaster* sp. | RS9009 | Lucanidae sp. | Sulawesi | RS |
| *Oigolaimella attenuata* | SB353 | *Reticulitermes lucifugus* | Corsica, France | SB |
| *Oigolaimella* sp. | RS9010 | *Reticulitermes speratus* | Kagoshima, Japan | RS/NK |
| *Parapristionchus giblindavisi* | RS5555 | *Dorcus rubrofemoratus* | Japan | RS |
| *Parasitodiplogaster maxinema* | nc | Syconium, *Ficus maxima* | Panama | (2) |
| *Paroigolaimella micura* | nc | Cow dung | Tübingen, Germany | RS |
| *Paroigolaimella stresemanni* | nc | Cow dung | Tübingen, Germany | RS |
| *Pristionchus elegans* | RS5229 | *Phleotrupes auratus* | Japan | RS |
| *Pristionchus fissidentatus* | RS5133 | Soil | Nepal | RS |
| *Pristionchus maupasi* | RS0143 | *Melolontha melolontha* | Tübingen, Germany | RS |
| *Pristionchus pacificus* | PS312 | Soil | Pasadena, CA, USA | RS |
| *Pristionchus uniformis* | RS0141 | *Melolontha melolontha* | Menz, Germany | RS |
| *Pseudodiplogasteroides* sp. | SB257 | Rotten cacti | Arizona, USA | SB |
| *Rhabditidoides* sp. | RS5443 | *Geotrupes* sp. | Tübingen, Germany | RS |
| *Rhabditolaimus* sp. 1 | RSA134 | *Euplatypus parallelus* | La Réunion Island | RS |
| *Rhabditolaimus* sp. 2 | RS5442 | Prioninae sp*.* | Ghana | RS |
| *Sachsia zurstrasseni* | nc | Cow dung | Tübingen, Germany | RS |
| *Sudhausia aristotokia* | RS9011 | *Onthophagus* sp. | Ghana | RS |
| *Sudhausia crassa* | RS9012 | *Onthophagus* sp. | Pretoria, South Africa | RS |
| *Teratodiplogaster* sp. 1 | nc | Syconium, *Ficus variegata* | Okinawa, Japan | RS/NK |
| *Teratodiplogaster* sp. 2 | nc | Syconium, *Ficus* sp. | Vietnam | RS |
| *Tylopharynx foetida* | nc | *Geotrupes* sp. | Corsica | RS |
| Non-diplogastrid Rhabditina and outgroups | | | | |
| *Brevibucca saprophaga* | SB261 | - | - | (3) |
| *Bunonema reticulatum* | RS9013 | Moss | Tübingen, Germany | RS |
| *Bunonema* sp. | RS9014 | - | New Caledonia | RS |
| *Caenorhabditis angaria* | PS1010 | *Metamasius hemipterus* | Florida, USA | (4) |
| *Caenorhabditis briggsae* | AF16 | Soil | Ahmedabad, India | CGC |
| *Caenorhabditis elegans* | N2 | Mushroom compost | Bristol, UK | CGC |
| *Choriorhabditis cristata* | RS5618 | Basidiomycota mushroom | Tübingen, Germany | RS |
| *Cruznema tripartitum* | - | Soil | Campillo de Arenas, Spain | (5) |
| *Diploscapter* sp. | RS1965 | Compost | Tübingen, Germany | RS |
| *Distolabrellus veechi* | - | Decaying organic substrate | India | (6) |
| *Heterorhabditis bacteriophora* | - | *Heliothis punctigera* | South Australia | CGC |
| *Mesorhabditis acidophila* | - | Snottites | Tabasco, Mexico | (7) |
| *Myolaimus byersi* | RGD233 | *Hyophorbe verschaffeltii* | Florida, USA | (8) |
| *Odontopharynx longicaudata* | - | Soil | Netherlands | (9) |
| *Oscheius carolinensis* | - | Vermicompost | North Carolina, USA | (10) |
| *Oscheius guentheri* | - | Decaying rice plants | Hau Giang, Vietnam | (11) |
| *Oscheius myriophila* | RS1964 | Soil | Oahu, HI, USA | RS |
| *Oscheius tipulae* | RS1990 | - | Bogota, Colombia | RS |
| *Panagrellus redivivus* | PS1163 | - | Harpenden, UK | CGC |
| *Parasitorhabditis obtusa* | RS5586 | *Ips typographus* | Tübingen, Germany | RS |
| *Pelodera cylindrica* | RS5097 | *Geotrupes* sp. | Tübingen, Germany | RS |
| *Pelodera strongyloides* | DF5022 | Cow skin | Illinois, USA | CGC |
| *Pelodera teres* | - | Manure | Kashmir, India | (12) |
| *Pellioditis* sp. | RS5624 | Basidiomycota mushroom | Tübingen, Germany | RS |
| *Poikilolaimus floridensis* | RGD617R | *Neotermes jouteli* | Florida, USA | (13) |
| *Poikilolaimus oxycercus* | SB200 | Compost | Belgium | SB |
| *Protorhabditis* sp. | RS9015 | Frass from *Dorcus rectus* | Tsukuba, Japan | RS |
| *Rhabditis brassicae* | RS5617 | Basidiomycota mushroom | Tübingen, Germany | RS |
| *Rhabditis colombiana* | - | *Cyrtomenus bergi* | Cauca Valley, Colombia | (14) |
| *Rhabditis rainai* | - | *Coptotermes formosanus* | Louisiana, USA | (15) |
| *Rhabditoides inermis* | SB328 | - | - | SB |
| *Strongyloides ratti* | ED321 | *Rattus norvegicus* | Philadelphia, USA | MV |
| *Teratorhabditis mariannae* | RS0136 | - | Tübingen, Germany | RS |
| *Teratorhabditis palmarum* | - | *Rhynchophorus palmarum* | Trinidad | (16) |
| *Teratorhabditis synpapillata* | - | *Rhynchophorus ferrugineus* | Kagoshima, Japan | (17) |
| *Zeldia punctata* | JB15 | Soil | Riverside, CA, USA | JB |

**Supplementary references for Figure 2-source data 1:**

1. Kanzaki N, Masuya H, Kubono T. 2008. Description of *Neodiplogaster crenatae* sp. n. and *N. acaloleptae* sp. n. (Nematoda: Diplogastridae) from Japan. *Nematology* **10**:545-560.
2. Kanzaki N, Giblin-Davis RM, Ye WM, Herre EA, Center BJ. 2013 Description of *Parasitodiplogaster pharmaconema* n. sp. and redescription of *P. maxinema* from *Ficus maxima* Mill. (Moraceae). *Nematology* **15**:957-974.
3. De Ley P, De Ley IT, Morris K, Abebe E, Mundo-Ocampo M, Yoder M, Heras J, Waumann D, Rocha-Olivares A, Burr AHJ, Baldwin JG, Thomas WK. 2005. An integrated approach to fast and informative morphological vouchering of nematodes for applications in molecular barcoding. *Phil Trans R Soc B* **360**:1945-1958.
4. Sudhaus W, Kiontke K, Giblin-Davis RM. 2010. Description of *Caenorhabditis angaria* n. sp. (Nematoda: Rhabditidae), an associate of sugarcane and palm weevils (Coleoptera: Curculionidae). *Nematology* **13**:61-78.
5. Abolafia J, Peña-Santiago R. 2009. Nematodes of the Order Rhabditida from Andalucía Oriental, Spain. The family Mesorhabditidae, with description of *Mesorhabditis carmenae* n. sp. *J Nematode Morphol Syst* **12**:41-64.
6. Tahseen Q, Shah AA, Khan R, Sultana R, Hussain A, Ahmad I. 2009. Biogeography and variations in allopatric populations of *Distolabrellus veechi* Anderson, 1983 (Nematoda: Rhabditidae). *Nematology* **11**:815-826.
7. Borgonie G, Dierick M, Houthoofd W, Willems M, Jacobs P, Bert W. 2010. Refuge from predation, the benefit of living in an extreme acidic environment? *Biol Bull* **219**:268-276.
8. Giblin-Davis RM, Kanzaki N, De Ley P, Williams DS, Schierenberg E, Ragsdale EJ, Zeng YS, Center BJ. 2010. Ultrastructure and life history of *Myolaimus byersi* n. sp. (Myolaimina: Myolaimidae), a phoretic associate of the crane fly, *Limonia schwarzi* (Alexander) (Limoniidae), in Florida. *Nematology* **12**:519-542.
9. Ye WM, Torres-Barragan A, Cardoza YJ. 2010. *Oscheius carolinensis* n. sp. (Nematoda: Rhabditidae), a potential entomopathogenic nematode from vermicompost. *Nematology* **12**:121-135.
10. van Megen H, van den Elsen S, Holterman M, Karssen G, Mooyman P, Bongers T, Holovachov O, Bakker J, Helder J. 2009. A phylogenetic tree of nematodes based on about 1200 full-length small subunit ribosomal DNA sequences. *Nematology* **11**:927-950.
11. Sudhaus W, Hooper DJ. 1990. *Rhabditis (Oscheius) guentheri* sp. n., an unusual species with reduced posterior ovary, with observations on the *Dolichura* and *Insectivora* groups (Nematoda: Rhabditidae). *Nematologica* **40**:508-533.
12. Hussain A, Tahseen Q, Khan R. 2006. Re-description of two species of *Pelodera* (Nematoda: Rhabditidae) from India. *Nematol Mediterr* **34**:55-62.
13. Kanzaki N, Giblin-Davis RM, Scheffrahn RH, Center BJ. 2009. *Poikilolaimus floridensis* n. sp. (Rhabditida: Rhabditidae) associated with termites (Kalotermitidae). *Nematology* **11**:203-216.
14. Stock SP, Caicedo AM, Calatayud PA. 2005. *Rhabditis (Oscheius) colombiana* n. sp. (Nematoda: Rhabditidae), a necromenic associate of the subterranean burrower bug *Cyrtomenus bergi* (Hemiptera: Cydnidae) from the Cauca Valley, Colombia. *Nematology* **7**:363-373.
15. Carta LK, Osbrink W. 2005. *Rhabditis rainai* n. sp. (Nematoda: Rhabditida) associated with the Formosan subterranean termite, *Coptotermes formosanus* (Isoptera: Rhinotermitidae). *Nematology* **7**:863-879.
16. Gerber K, Giblin-Davis RM. 1990. *Teratorhabditis palmarum* n. sp. (Nematoda: Rhabditidae): an associate of *Rhynchophorus palmarum* and *R. cruentatus*. *J Nematol* **22**:337-347.
17. Kanzaki N, Abe F, Giblin-Davis RM, Kiontke K, Fitch DHA, Hata K, Soné K. 2008. *Teratorhabditis synpapillata* Sudhaus, 1985 (Rhabditida: Rhabditidae) is an associate of the red palm weevil, *Rhynchophorus ferrugineus* (Coleoptera: Curculionidae). *Nematology* **10**:207-218.

**Figure 2-source data 1b. GenBank accession numbers for gene sequences analyzed in this study.** Sequences shorter than 200 bp (with accession numbers beginning with “VS”) are available at www.pristionchus.org/download/suppSusoy2014.html.

| Nematode species | 18s rRNA | 28S rRNA | *rpl-2* | *rpl-6* | *rpl-9* | *rpl-10* | *rpl-14* | *rpl-23* | *rpl-35* | *rps-7* | *rps-14* | *rps-27* |  | *RNAp2* |
| --- | --- | --- | --- | --- | --- | --- | --- | --- | --- | --- | --- | --- | --- | --- |
| *Acrostichus halicti* | AB455817 | EU195983 | - | - | - | - | - | - | - | - | - | - | - | - |
| *Acrostichus* cf. *nudicapitatus* | JX163980 | KJ877318 | GQ422215 | Seq | GQ422185 | GQ422200 | GQ422323 | GQ422338 | GQ422353 | GQ422248 | GQ422278 | GQ422293 |  | - |
| *Acrostichus rhynchophori* | AB455210 | KJ877244 | KJ877294 | KJ877312 | KJ877327 | KJ877343 | KJ877359 | KJ877374 | KJ877391 | KJ877407 | KJ877424 | KJ877441 | VS0001 | - |
| *Allodiplogaster hylobii* | KJ877224 | KJ877266 | - | - | - | - | - | - | - | - | - | - | - | - |
| *Allodiplogaster* sp. 1 | JX163979 | JX163970 | EF634530 | GQ422165 | GQ422183 | EF634549 | EF634568 | EF634606 | EF634796 | GQ422246 | EF634891 | EF634986 | EF635005 | - |
| *Allodiplogaster sudhausi* | KJ877226 | KJ877267 | GQ422219 | GQ422171 | GQ422189 | GQ422204 | GQ422327 | GQ422342 | GQ422357 | GQ422252 | GQ422282 | GQ422297 |  |  |
| *Butlerius* sp. | KJ877204 | KJ877247 | - | - | - | - | - | - | - | - | - | - | - | - |
| *Diplogasteriana* sp. | KJ877202 | KJ877245 | - | - | - | - | - | - | - | - | - | - | - | - |
| *Diplogasteriana schneideri* | KJ877203 | KJ877246 | GQ422208 | GQ422157 | GQ422175 | GQ422193 | GQ422316 | GQ422331 | GQ422346 | GQ422238 | GQ422271 | GQ422286 |  | - |
| *Diplogasteroides magnus* | KJ877214 | KJ877270 | GQ422214 | GQ422166 | GQ422184 | GQ422199 | GQ422322 | GQ422337 | GQ422352 | GQ422247 | GQ422277 | GQ422292 |  | - |
| *Diplogasteroides* sp. 1 | KJ877215 | KJ877271 | GQ422209 | GQ422158 | GQ422176 | GQ422194 | GQ422317 | GQ422332 | GQ422347 | GQ422239 | GQ422272 | GQ422287 |  | - |
| *Diplogastrellus gracilis* | KJ877216 | KJ877249 | GQ422217 | GQ422169 | GQ422187 | GQ422202 | GQ422325 | GQ422340 | GQ422355 | GQ422250 | GQ422280 | GQ422295 |  | - |
| *Diplogastrellus* sp. | KJ877205 | KJ877248 | KJ877295 | KJ877313 | KJ877328 | KJ877344 | KJ877360 | KJ877375 | KJ877392 | KJ877408 | KJ877425 | KJ877442 | VS0002 | - |
| *Eudiplogasterium levidentum* | KJ877206 | KJ877258 | - | - | - | - | - | - | - | - | - | - | - | - |
| *Fictor* sp. 1 | KJ877233 | KJ877280 | KJ877288 | KJ877304 | KJ877321 | KJ877337 | KJ877353 | KJ877368 | KJ877384 | KJ877400 | KJ877418 | KJ877434 | VS0003 | - |
| *Fictor* sp. 2 | KJ877234 | KJ877281 | KJ877289 | KJ877306 | KJ877322 | KJ877338 | KJ877354 | KJ877369 | KJ877385 | KJ877401 | KJ877419 | KJ877435 | VS0004 | - |
| *Fictor stercorarius* | KJ877235 | KJ877282 | KJ877290 | KJ877308 | KJ877323 | KJ877339 | KJ877356 | KJ877370 | KJ877387 | KJ877403 | - | KJ877437 | VS0005 | - |
| *Fuchsnema halleri* | KJ877227 | KJ877253 | KJ877292 | KJ877310 | KJ877325 | KJ877341 | KJ877357 | KJ877372 | KJ877389 | KJ877405 | KJ877422 | KJ877439 | VS0006 | - |
| *Fuchsnema* sp. | KJ877228 | KJ877254 | KJ877299 | KJ877316 | KJ877332 | KJ877348 | KJ877364 | KJ877379 | KJ877395 | KJ877412 | KJ877429 | KJ877445 | VS0007 | - |
| *Koerneria luziae* | AB597232 | KJ877284 | KJ877291 | KJ877309 | KJ877324 | KJ877340 | - | KJ877371 | KJ877388 | KJ877404 | KJ877421 | KJ877438 | VS0008 | - |
