# Supplementary material for: Rapid diversification associated with a macroevolutionary pulse of developmental plasticity
Source: eLife. 2015 Feb 4;4:e05463. doi: 10.7554/eLife.05463 (PMC4357287; doi:10.7554/eLife.05463)
Supplement: Figure 3—source data 1. — (A) Results of principal component analysis of stomatal form in Rhabditina, including both morphs of dimorphic taxa. Loadings of Procrustes coordinates and log centroid size of stomatal form onto the first four principal component (PC) axes are shown, as are the proportion of variance explained by those PC axes (B) Results of principal component analysis of stomatal shape in Rhabditina, including both morphs of dimorphic taxa. Loadings of Procrustes coordinates of stomatal shape onto the first four principal component (PC) axes are shown, as are the proportion of variance explained by those PC axes. (C) Results of phylogenetic principal component analysis of stomatal form in Rhabditina, with dimorphic taxa represented by the stenostomatous (St) morph. Loadings of Procrustes coordinates and log centroid size of stomatal form onto the first four principal component (PC) axes are shown, as are the proportion of variance explained by those PC axes. (D) Results of phylogenetic principal component analysis of stomatal shape in Rhabditina, with dimorphic taxa represented by the stenostomatous (St) morph. Loadings of Procrustes coordinates of stomatal shape onto the first four principal component (PC) axes are shown, as are the proportion of variance explained by those PC axes. (E) Estimates of morphological disparity of the stoma in Rhabditina. Groups compared were non-diplogastrid Rhabditina, monomorphic Diplogastridae, and dimorphic Diplogastridae. Disparity was measured as the principal component (PC) analysis volume and the sum of univariate variances. PC scores along the first two and three PC axes of Procrustes form and shape space, respectively, were used and are presented in the form mean ± standard deviation (95% confidence interval). Eu, eurystomatous; St, stenostomatous. (F) Rates of stomatal evolution along the first principal component (PC) axis of Procrustes form- and shape-space. Rates were compared for non-diplogastrid Rhabditina (Rh), dimorphic Diplogastri [file elife05463s003.docx]

**Figure 3-source data** **1a. Results of principal component analysis ofstomatal form in Rhabditina, including both morphs of dimorphic taxa.** Loadings of Procrustes coordinates and log centroid size of stomatal form onto the first four principal component (PC) axes are shown, as are the proportion of variance explained by those PC axes.

|  | PC1 | PC2 | PC3 | PC4 |
| --- | --- | --- | --- | --- |
| Procrustes coordinate 1 | -0.052 | 0.101 | -0.074 | -0.216 |
| Procrustes coordinate 2 | -0.052 | 0.192 | 0.150 | -0.011 |
| Procrustes coordinate 3 | -0.086 | 0.223 | -0.020 | -0.077 |
| Procrustes coordinate 4 | -0.143 | 0.246 | 0.011 | -0.119 |
| Procrustes coordinate 5 | -0.054 | 0.168 | -0.058 | -0.113 |
| Procrustes coordinate 6 | -0.107 | 0.180 | -0.024 | -0.192 |
| Procrustes coordinate 7 | -0.103 | 0.225 | 0.067 | -0.102 |
| Procrustes coordinate 8 | 0.009 | 0.024 | -0.561 | 0.305 |
| Procrustes coordinate 9 | -0.049 | 0.094 | -0.061 | -0.132 |
| Procrustes coordinate 10 | 0.054 | -0.160 | 0.363 | 0.162 |
| Procrustes coordinate 11 | 0.008 | -0.015 | -0.064 | -0.036 |
| Procrustes coordinate 12 | 0.061 | -0.161 | 0.453 | 0.226 |
| Procrustes coordinate 13 | -0.059 | 0.123 | 0.043 | 0.249 |
| Procrustes coordinate 14 | 0.224 | -0.469 | -0.165 | -0.654 |
| Procrustes coordinate 15 | 0.113 | -0.285 | 0.115 | 0.016 |
| Procrustes coordinate 16 | 0.098 | -0.099 | -0.471 | 0.337 |
| Procrustes coordinate 17 | 0.084 | -0.210 | 0.049 | 0.154 |
| Procrustes coordinate 18 | -0.061 | 0.060 | 0.018 | -0.102 |
| Procrustes coordinate 19 | 0.134 | -0.305 | -0.003 | 0.079 |
| Procrustes coordinate 20 | -0.069 | 0.073 | 0.045 | -0.035 |
| Procrustes coordinate 21 | 0.064 | -0.119 | 0.005 | 0.176 |
| Procrustes coordinate 22 | -0.014 | 0.115 | 0.182 | 0.082 |
| Log centroid size | 0.905 | 0.414 | 0.039 | -0.022 |
| Proportion of variance | 0.73 | 0.16 | 0.05 | 0.03 |
| Cumulative proportion | 0.73 | 0.88 | 0.93 | 0.96 |

**Figure 3-source data 1b. Results of principal component analysis of stomatal shape in Rhabditina, including both morphs of dimorphic taxa.** Loadings of Procrustes coordinates of stomatal shape onto the first four principal component (PC) axes are shown, as are the proportion of variance explained by those PC axes.

|  | PC1 | PC2 | PC3 | PC4 | PC5 |
| --- | --- | --- | --- | --- | --- |
| Procrustes coordinate 1 | -0.12 | 0.07 | -0.22 | -0.11 | -0.40 |
| Procrustes coordinate 2 | -0.17 | -0.12 | 0.02 | 0.44 | -0.18 |
| Procrustes coordinate 3 | -0.22 | 0.04 | -0.06 | 0.26 | 0.04 |
| Procrustes coordinate 4 | -0.31 | -0.01 | -0.12 | -0.20 | 0.39 |
| Procrustes coordinate 5 | -0.16 | 0.07 | -0.10 | 0.16 | -0.23 |
| Procrustes coordinate 6 | -0.23 | 0.01 | -0.20 | -0.34 | -0.18 |
| Procrustes coordinate 7 | -0.25 | -0.05 | -0.09 | 0.16 | 0.13 |
| Procrustes coordinate 8 | 0.00 | 0.57 | 0.28 | -0.04 | 0.16 |
| Procrustes coordinate 9 | -0.11 | 0.06 | -0.13 | 0.00 | 0.02 |
| Procrustes coordinate 10 | 0.15 | -0.37 | 0.17 | -0.06 | -0.14 |
| Procrustes coordinate 11 | 0.02 | 0.06 | -0.04 | -0.03 | -0.02 |
| Procrustes coordinate 12 | 0.16 | -0.45 | 0.23 | -0.03 | 0.01 |
| Procrustes coordinate 13 | -0.14 | -0.04 | 0.25 | -0.15 | 0.30 |
| Procrustes coordinate 14 | 0.52 | 0.13 | -0.66 | 0.20 | 0.26 |
| Procrustes coordinate 15 | 0.29 | -0.13 | 0.01 | -0.18 | -0.11 |
| Procrustes coordinate 16 | 0.18 | 0.48 | 0.33 | 0.06 | -0.27 |
| Procrustes coordinate 17 | 0.22 | -0.06 | 0.15 | -0.04 | 0.29 |
| Procrustes coordinate 18 | -0.11 | -0.03 | -0.11 | -0.30 | -0.17 |
| Procrustes coordinate 19 | 0.33 | -0.01 | 0.07 | -0.16 | -0.26 |
| Procrustes coordinate 20 | -0.12 | -0.06 | -0.05 | -0.22 | 0.16 |
| Procrustes coordinate 21 | 0.14 | 0.00 | 0.18 | 0.08 | 0.25 |
| Procrustes coordinate 22 | -0.08 | -0.15 | 0.11 | 0.49 | -0.03 |
| Proportion of variance | 0.68 | 0.12 | 0.09 | 0.05 | 0.01 |
| Cumulative proportion | 0.68 | 0.81 | 0.90 | 0.94 | 0.96 |

**Figure 3-source data 1c. Results of phylogenetic principal component analysis of stomatal form in Rhabditina, with dimorphic taxa represented by the stenostomatous (St) morph.** Loadings of Procrustes coordinates and log centroid size of stomatal form onto the first four principal component (PC) axes are shown, as are the proportion of variance explained by those PC axes.

|  | PC1 | PC2 | PC3 | PC4 |
| --- | --- | --- | --- | --- |
| Procrustes coordinate 1 | -0.360 | -0.726 | 0.063 | 0.092 |
| Procrustes coordinate 2 | -0.060 | -0.280 | 0.245 | -0.777 |
| Procrustes coordinate 3 | -0.362 | -0.808 | -0.079 | -0.301 |
| Procrustes coordinate 4 | -0.515 | -0.698 | 0.165 | 0.289 |
| Procrustes coordinate 5 | -0.352 | -0.832 | -0.076 | -0.146 |
| Procrustes coordinate 6 | -0.442 | -0.678 | 0.124 | 0.435 |
| Procrustes coordinate 7 | -0.450 | -0.796 | 0.122 | -0.102 |
| Procrustes coordinate 8 | 0.211 | -0.216 | -0.816 | -0.200 |
| Procrustes coordinate 9 | -0.470 | -0.638 | -0.085 | 0.089 |
| Procrustes coordinate 10 | 0.180 | 0.769 | 0.549 | -0.070 |
| Procrustes coordinate 11 | 0.259 | -0.030 | -0.176 | 0.351 |
| Procrustes coordinate 12 | 0.180 | 0.687 | 0.648 | -0.121 |
| Procrustes coordinate 13 | -0.172 | -0.060 | 0.373 | -0.186 |
| Procrustes coordinate 14 | 0.336 | 0.537 | -0.446 | 0.333 |
| Procrustes coordinate 15 | 0.380 | 0.866 | 0.044 | 0.064 |
| Procrustes coordinate 16 | 0.489 | 0.134 | -0.681 | -0.265 |
| Procrustes coordinate 17 | 0.412 | 0.799 | -0.035 | -0.035 |
| Procrustes coordinate 18 | -0.490 | -0.399 | 0.190 | 0.528 |
| Procrustes coordinate 19 | 0.416 | 0.859 | -0.054 | 0.141 |
| Procrustes coordinate 20 | -0.482 | -0.421 | 0.320 | 0.346 |
| Procrustes coordinate 21 | 0.370 | 0.570 | -0.098 | 0.098 |
| Procrustes coordinate 22 | 0.169 | 0.047 | 0.235 | -0.842 |
| Log centroid size | 0.997 | -0.080 | 0.022 | 0.009 |
| Proportion of variance | 0.76 | 0.12 | 0.04 | 0.03 |
| Cumulative proportion | 0.76 | 0.87 | 0.92 | 0.94 |

**Figure 3-source data 1d. Results of phylogenetic principal component analysis of stomatal shape in Rhabditina, with dimorphic taxa represented by the stenostomatous (St) morph.** Loadings of Procrustes coordinates of stomatal shape onto the first four principal component (PC) axes are shown, as are the proportion of variance explained by those PC axes.

|  | PC1 | PC2 | PC3 | PC4 | PC5 |
| --- | --- | --- | --- | --- | --- |
| Procrustes coordinate 1 | -0.81 | 0.00 | 0.07 | 0.34 | 0.33 |
| Procrustes coordinate 2 | -0.27 | -0.12 | -0.80 | 0.47 | 0.08 |
| Procrustes coordinate 3 | -0.87 | 0.18 | -0.30 | 0.14 | -0.19 |
| Procrustes coordinate 4 | -0.88 | -0.16 | 0.26 | -0.12 | -0.24 |
| Procrustes coordinate 5 | -0.89 | 0.17 | -0.15 | 0.22 | 0.13 |
| Procrustes coordinate 6 | -0.82 | -0.12 | 0.40 | -0.19 | 0.19 |
| Procrustes coordinate 7 | -0.92 | -0.05 | -0.13 | -0.02 | -0.20 |
| Procrustes coordinate 8 | -0.03 | 0.89 | -0.12 | -0.25 | 0.12 |
| Procrustes coordinate 9 | -0.78 | 0.08 | 0.11 | 0.19 | 0.07 |
| Procrustes coordinate 10 | 0.72 | -0.63 | -0.11 | -0.03 | 0.11 |
| Procrustes coordinate 11 | 0.11 | 0.22 | 0.32 | -0.06 | 0.10 |
| Procrustes coordinate 12 | 0.65 | -0.70 | -0.18 | -0.13 | -0.02 |
| Procrustes coordinate 13 | -0.16 | -0.36 | -0.23 | -0.34 | 0.03 |
| Procrustes coordinate 14 | 0.65 | 0.35 | 0.41 | 0.51 | -0.14 |
| Procrustes coordinate 15 | 0.93 | -0.14 | 0.09 | 0.08 | 0.10 |
| Procrustes coordinate 16 | 0.41 | 0.77 | -0.22 | -0.32 | 0.05 |
| Procrustes coordinate 17 | 0.90 | -0.03 | -0.02 | -0.21 | -0.21 |
| Procrustes coordinate 18 | -0.61 | -0.27 | 0.51 | -0.15 | 0.31 |
| Procrustes coordinate 19 | 0.95 | -0.04 | 0.17 | -0.04 | 0.20 |
| Procrustes coordinate 20 | -0.63 | -0.37 | 0.32 | -0.14 | -0.34 |
| Procrustes coordinate 21 | 0.68 | 0.06 | 0.10 | -0.40 | -0.36 |
| Procrustes coordinate 22 | 0.13 | -0.11 | -0.87 | 0.40 | -0.10 |
| Proportion of variance | 0.52 | 0.17 | 0.10 | 0.07 | 0.03 |
| Cumulative proportion | 0.52 | 0.69 | 0.79 | 0.86 | 0.90 |

**Figure 3-source data 1e. Estimates of morphological disparity of the stoma in Rhabditina.** Groups compared were non-diplogastrid Rhabditina, monomorphic Diplogastridae, and dimorphic Diplogastridae. Disparity was measured as the principal component (PC) analysis volume and the sum of univariate variances. PC scores along the first two and three PC axes of Procrustes form and shape space, respectively, were used and are presented in the form mean±standard deviation (95% confidence interval). Eu, eurystomatous; St, stenostomatous.

| Procrustes form-space | | | | |
| --- | --- | --- | --- | --- |
| Group | n | Rarefaction | Sum of variances | PCA volume |
| Non-diplogastrid Rhabditina | 33 | 23 | 0.09±0.025  (0.046-0.144) | 0.066±0.021  (0.032-0.112) |
| Monomorphic and St Diplogastridae | 54 | 23 | 0.241±0.06  (0.135-0.367) | 0.163±0.053  (0.078-0.279) |
| Monomorphic, Eu, and St Diplogastridae | 77 | 23 | 0.218±0.041  (0.145-0.305) | 0.13±0.032  (0.081-0.204) |
| St Diplogastridae | 23 | 23 | 0.097±0.031  (0.043-0.166) | 0.061±0.021  (0.027-0.109) |
| Eu Diplogastridae | 23 | 23 | 0.068±0.027  (0.026-0.129) | 0.044±0.02  (0.015-0.092) |
| Monomorphic Diplogastridae | 31 | 23 | **0.301±0.083**  **(0.151-0.475)** | **0.219±0.074**  **(0.099-0.383)** |
| Procrustes shape-space | | | | |
| Group | n | Rarefaction | Sum of variances | PCA volume |
| Non-diplogastrid Rhabditina | 33 | 23 | 0.0385±0.0045  (0.0297-0.0475) | 0.0291±0.003  (0.023-0.0349) |
| Monomorphic and St Diplogastridae | 54 | 23 | 0.0749±0.0082  (0.0584-0.0905) | 0.0536±0.0076  (0.0386-0.0683) |
| Monomorphic, Eu, and St Diplogastridae | 77 | 23 | 0.0705±0.0084  (0.0532-0.0865) | 0.0513±0.0077  (0.0361-0.0661) |
| St Diplogastridae | 23 | 23 | 0.0342±0.0132  (0.0117-0.0618) | 0.0227±0.0107  (0.0063-0.0463) |
| Eu Diplogastridae | 23 | 23 | 0.0205±0.0094  (0.0064-0.0411) | 0.014±0.008  (0.0044-0.0329) |
| Monomorphic Diplogastridae | 31 | 23 | **0.0781±0.0105**  **(0.0575-0.0987)** | **0.0566±0.0093**  **(0.0386-0.0750)** |

**Figure 3-source data 1f. Rates of stomatal evolution along the first principal component (PC) axis of Procrustes form- and shape-space.** Rates were compared for non-diplogastrid Rhabditina (Rh), dimorphic Diplogastridae (Dm), and monomorphic Diplogastridae (Mn). Numbers indicate separate rate parameters for the designated groups. Model-averaged rates with standard deviation are shown.

| Procrustes form-space | | | | | | |
| --- | --- | --- | --- | --- | --- | --- |
| Model (Rh-Dm-Mn) | Rate 1 | Rate 2 | Rate 3 | -lnL | *k* | AICc |
| 1-1-2 | 0.378±0.127 | 1.292±0.201 | na | -38.682 | 3 | **83.37** |
| 1-2-3 | 0.340±0.124 | 0.438±0.136 | 1.273±0.202 | -38.409 | 4 | 84.83 |
| 1-2-2 | 0.347±0.120 | 0.831±0.165 | na | -41.330 | 3 | 88.67 |
| 1-1-1 | 0.644±0.145 | na | na | -45.001 | 2 | 94.01 |
| 1-2-1 | 0.693±0.156 | 0.548±0.134 | na | -44.073 | 3 | 94.15 |
| Procrustes shape-space | | | | | | |
| Model (Rh-Dm-Mn) | Rate 1 | Rate 2 | Rate 3 | -lnL | *k* | AICc |
| 1-2-3 | 0.044±0.012 | 0.094±0.029 | 0.244±0.06 | 38.107 | 4 | **-68.20** |
| 1-1-2 | 0.064±0.016 | 0.247±0.060 | na | 36.514 | 3 | -67.02 |
| 1-2-2 | 0.042±0.011 | 0.169±0.039 | na | 35.585 | 3 | -65.16 |
| 1-1-1 | 0.118±0.027 | na | na | 28.711 | 2 | -53.41 |
| 1-2-1 | 0.125±0.031 | 0.104±0.026 | na | 29.468 | 3 | -52.93 |

**Figure 3-source data 1g. Statistical comparison of non-nested models of stomatal evolution along the first principal component (PC) axis of Procrustes form- and shape-space using a chi-square distribution.** Numbers indicate separate rate parameters for the designated groups. Rh, non-diplogastrid Rhabditina (Rh); Dm, dimorphic Diplogastridae; Mn, monomorphic Diplogastridae.

|  | Procrustes form-space |  |
| --- | --- | --- |
| Null model  (Rh-Dm-Mn) | Alternative model  (Rh-Dm-Mn) | Chi-square p-values |
| 1-1-1 | 1-2-1 | 0.2435 |
| 1-1-1 | 1-2-2 | **0.0108** |
| 1-1-1 | 1-1-2 | **0.0011** |
| 1-1-1 | 1-2-3 | **0.0038** |
| 1-2-1 | 1-2-3 | **0.0058** |
| 1-2-2 | 1-2-3 | 0.0923 |
| 1-1-2 | 1-2-3 | 0.7931 |
|  | Procrustes shape-space |  |
| Null model  (Rh-Dm-Mn) | Alternative model  (Rh-Dm-Mn) | Chi-square p-values |
| 1-1-1 | 1-2-1 | 0.3236 |
| 1-1-1 | 1-2-2 | **0.0007** |
| 1-1-1 | 1-1-2 | **0.0002** |
| 1-1-1 | 1-2-3 | **0.0002** |
| 1-2-1 | 1-2-3 | **0.0005** |
| 1-2-2 | 1-2-3 | 0.1314 |
| 1-1-2 | 1-2-3 | 0.2968 |

**Figure 3-source data 1h. Highest posterior densities (HPD) of rates, and associated p-values obtained from two-tailed randomization tests, of stomatal form and shape evolution.** Tests were performed for the branches assigned to non-diplogastrid Rhabditina, dimorphic Diplogastridae, and monomorphic Diplogastridae. CI, confidence interval.

| Procrustes form-space | | | | |
| --- | --- | --- | --- | --- |
| Lineage | HPD mean  (95% CI) | Non-diplogastrid Rhabditina | Dimorphic Diplogastridae | Monomorphic Diplogastridae |
| Non-diplogastrid Rhabditina | 0.727  (0.117-1.482) | 0.998 |  |  |
| Dimorphic Diplogastridae | 1.402  (0.251-2.807) | 0.391 | 0.987 |  |
| Monomorphic Diplogastridae | 3.079  (0.606-7.335) | **0.041** | 0.336 | 0.997 |
| Procrustes shape-space | | | | |
| Lineage | HPD mean  (95% CI) | Non-diplogastrid Rhabditina | Dimorphic Diplogastridae | Monomorphic Diplogastridae |
| Non-diplogastrid Rhabditina | -1.074  (-1.523--0.560) | 0.998 |  |  |
| Dimorphic Diplogastridae | -0.358  (-0.577--0.145) | **0.017** | 0.998 |  |
| Monomorphic Diplogastridae | -0.282  (-0.525--0.053) | **0.012** | 0.652 | 0.998 |
