# Supplementary material for: Rapid diversification associated with a macroevolutionary pulse of developmental plasticity
Source: eLife. 2015 Feb 4;4:e05463. doi: 10.7554/eLife.05463 (PMC4357287; doi:10.7554/eLife.05463)
Supplement: Figure 4—source data 1. — Structures were recorded as described in text. Presence/absence of dimorphism is also given. 0 = absence, 1 = presence. DOI: http://dx.doi.org/10.7554/eLife.05463.014 [file elife05463s004.docx]

**Figure 4-source data 1. Matrix of structures tabulated to measure stomatal complexity.** Structures were recorded as described in text. Presence/absence of dimorphism is also given. 0 = absence, 1 = presence.

|  | *Acrostichus* cf. *nudicapitatus* | *Acrostichus halicti* | *Acrostichus rhynchophori* | *Allodiplogaster hylobii* | *Allodiplogaster* sp. 1 | *Allodiplogaster sudhausi* | *Brevibucca saprophaga* | *Bunonema reticulatum* | *Bunonema* sp. | *Butlerius* sp. | *Caenorhabditis angaria* | *Caenorhabditis briggsae* |
| --- | --- | --- | --- | --- | --- | --- | --- | --- | --- | --- | --- | --- |
| **Complexity index** | **3** | **4** | **3** | **5** | **6** | **5** | **0** | **0** | **0** | **3** | **1** | **1** |
| Glottoid apparatus^a^ | 0 | 0 | 0 | 0 | 0 | 0 | 0 | 0 | 0 | 0 | 1 | 1 |
| Moveable dorsal tooth^a^ | 1 | 1 | 1 | 1 | 1 | 1 | 0 | 0 | 0 | 1 | 0 | 0 |
| Fixed dorsal tooth^b^ | 0 | 0 | 0 | 0 | 0 | 0 | 0 | 0 | 0 | 0 | 0 | 0 |
| Serratae on anterior gymnostom* | 0 | 0 | 0 | 0 | 1 | 0 | 0 | 0 | 0 | 0 | 0 | 0 |
| Pro-/mesostegomatal serratae* | 0 | 0 | 0 | 0 | 0 | 0 | 0 | 0 | 0 | 0 | 0 | 0 |
| Right subdorsal denticle^a^ | 0 | 0 | 0 | 0 | 0 | 0 | 0 | 0 | 0 | 0 | 0 | 0 |
| Right subventral tooth^a^ | 0 | 0 | 0 | 1 | 1 | 1 | 0 | 0 | 0 | 0 | 0 | 0 |
| Right subventral ridge of denticles^c^ | 0 | 0 | 0 | 0 | 0 | 0 | 0 | 0 | 0 | 0 | 0 | 0 |
| Left subventral denticle or ridge* | 1 | 1 | 1 | 1 | 1 | 1 | 0 | 0 | 0 | 0 | 0 | 0 |
| Paired subventral denticles^a^ | 0 | 0 | 0 | 0 | 0 | 0 | 0 | 0 | 0 | 1 | 0 | 0 |
| Duplicate left subventral ridge* | 0 | 0 | 0 | 0 | 0 | 0 | 0 | 0 | 0 | 0 | 0 | 0 |
| Right ventral denticle^c^ | 0 | 0 | 0 | 0 | 0 | 0 | 0 | 0 | 0 | 0 | 0 | 0 |
| Lateral denticles^a^ | 0 | 1 | 0 | 0 | 0 | 0 | 0 | 0 | 0 | 0 | 0 | 0 |
| Pairs of metastegostomatal rods^d^ | 0 | 0 | 0 | 0 | 0 | 0 | 0 | 0 | 0 | 0 | 0 | 0 |
| Subventral stegostomatal warts^a^ | 0 | 0 | 0 | 0 | 0 | 0 | 0 | 0 | 0 | 0 | 0 | 0 |
| Telostegostomatal ridge^e^ | 0 | 0 | 0 | 0 | 0 | 0 | 0 | 0 | 0 | 0 | 0 | 0 |
| Triadial telostegostomatal points^d^ | 0 | 0 | 0 | 0 | 0 | 0 | 0 | 0 | 0 | 0 | 0 | 0 |
| Dorsal, basal telostegostomatal fold* | 0 | 0 | 0 | 0 | 0 | 0 | 0 | 0 | 0 | 0 | 0 | 0 |
| Articulated apodemes^a^ | 0 | 0 | 0 | 1 | 1 | 1 | 0 | 0 | 0 | 0 | 0 | 0 |
| Perradial telostegostomatal knobs^a^ | 0 | 0 | 0 | 0 | 0 | 0 | 0 | 0 | 0 | 0 | 0 | 0 |
| Prostegostomatal row of denticles^b^ | 0 | 0 | 0 | 0 | 0 | 0 | 0 | 0 | 0 | 0 | 0 | 0 |
| Gymnostomatal denticles^b^ | 0 | 0 | 0 | 0 | 0 | 0 | 0 | 0 | 0 | 0 | 0 | 0 |
| Prostego-/gymnostomatal fold^e^ | 0 | 0 | 0 | 0 | 0 | 0 | 0 | 0 | 0 | 0 | 0 | 0 |
| Radial gymnostomatal divisions* | 0 | 0 | 0 | 0 | 0 | 0 | 0 | 0 | 0 | 0 | 0 | 0 |
| Radial cheilostomatal divisions^a^ | 1 | 1 | 1 | 1 | 1 | 1 | 0 | 0 | 0 | 1 | 0 | 0 |
| Stomatal dimorphism | 0 | 1 | 0 | 1 | 1 | 1 | 0 | 0 | 0 | 0 | 0 | 0 |

^a^See Fürst von Lieven and Sudhaus (2000).

^b^See Fürst von Lieven (2000).

^c^See Kanzaki et al. (2012).

^d^See Herrmann et al. (2013).

^e^See Ragsdale et al. (2014).

*Original in this study.

**Figure 4-source data 1, continued. Matrix of structures tabulated to measure stomatal complexity.** Structures were recorded as described in text. Presence/absence of dimorphism is also given. 0 = absence, 1 = presence.

|  | *Caenorhabditis elegans* | *Choriorhabditis cristata* | *Cruznema tripartitum* | *Diplogasteriana* n. sp. | *Diplogasteriana schneideri* | *Diplogasteroides magnus* | *Diplogasteroides* sp. | *Diplogastrellus gracilis* | *Diplogastrellus (Metadiplogaster)* sp. | *Diploscapter* sp. | *Distolabrellus veechi* | *Eudiplogasterium levidentum* |
| --- | --- | --- | --- | --- | --- | --- | --- | --- | --- | --- | --- | --- |
| **Complexity index** | **1** | **1** | **1** | **4** | **4** | **1** | **1** | **3** | **3** | **0** | **1** | **2** |
| Glottoid apparatus^a^ | 1 | 1 | 1 | 0 | 0 | 0 | 0 | 0 | 0 | 0 | 1 | 0 |
| Moveable dorsal tooth^a^ | 0 | 0 | 0 | 1 | 1 | 1 | 1 | 1 | 1 | 0 | 0 | 1 |
| Fixed dorsal tooth^b^ | 0 | 0 | 0 | 0 | 0 | 0 | 0 | 0 | 0 | 0 | 0 | 0 |
| Serratae on anterior gymnostom* | 0 | 0 | 0 | 0 | 0 | 0 | 0 | 0 | 0 | 0 | 0 | 0 |
| Pro-/mesostegomatal serratae* | 0 | 0 | 0 | 0 | 0 | 0 | 0 | 0 | 0 | 0 | 0 | 0 |
| Right subdorsal denticle^a^ | 0 | 0 | 0 | 0 | 0 | 0 | 0 | 0 | 0 | 0 | 0 | 0 |
| Right subventral tooth^a^ | 0 | 0 | 0 | 0 | 0 | 0 | 0 | 0 | 0 | 0 | 0 | 0 |
| Right subventral ridge of denticles^c^ | 0 | 0 | 0 | 0 | 0 | 0 | 0 | 0 | 0 | 0 | 0 | 0 |
| Left subventral denticle or ridge* | 0 | 0 | 0 | 1 | 1 | 0 | 0 | 0 | 0 | 0 | 0 | 0 |
| Paired subventral denticles^a^ | 0 | 0 | 0 | 0 | 0 | 0 | 0 | 1 | 1 | 0 | 0 | 0 |
| Duplicate left subventral ridge* | 0 | 0 | 0 | 0 | 0 | 0 | 0 | 0 | 0 | 0 | 0 | 0 |
| Right ventral denticle^c^ | 0 | 0 | 0 | 0 | 0 | 0 | 0 | 0 | 0 | 0 | 0 | 0 |
| Lateral denticles^a^ | 0 | 0 | 0 | 1 | 1 | 0 | 0 | 0 | 0 | 0 | 0 | 0 |
| Pairs of metastegostomatal rods^d^ | 0 | 0 | 0 | 0 | 0 | 0 | 0 | 0 | 0 | 0 | 0 | 0 |
| Subventral stegostomatal warts^a^ | 0 | 0 | 0 | 0 | 0 | 0 | 0 | 0 | 0 | 0 | 0 | 0 |
| Telostegostomatal ridge^e^ | 0 | 0 | 0 | 0 | 0 | 0 | 0 | 0 | 0 | 0 | 0 | 0 |
| Triadial telostegostomatal points^d^ | 0 | 0 | 0 | 0 | 0 | 0 | 0 | 0 | 0 | 0 | 0 | 0 |
| Dorsal, basal telostegostomatal fold* | 0 | 0 | 0 | 0 | 0 | 0 | 0 | 0 | 0 | 0 | 0 | 0 |
| Articulated apodemes^a^ | 0 | 0 | 0 | 0 | 0 | 0 | 0 | 0 | 0 | 0 | 0 | 0 |
| Perradial telostegostomatal knobs^a^ | 0 | 0 | 0 | 0 | 0 | 0 | 0 | 0 | 0 | 0 | 0 | 0 |
| Prostegostomatal row of denticles^b^ | 0 | 0 | 0 | 0 | 0 | 0 | 0 | 0 | 0 | 0 | 0 | 0 |
| Gymnostomatal denticles^b^ | 0 | 0 | 0 | 0 | 0 | 0 | 0 | 0 | 0 | 0 | 0 | 0 |
| Prostego-/gymnostomatal fold^e^ | 0 | 0 | 0 | 0 | 0 | 0 | 0 | 0 | 0 | 0 | 0 | 0 |
| Radial gymnostomatal divisions* | 0 | 0 | 0 | 0 | 0 | 0 | 0 | 0 | 0 | 0 | 0 | 0 |
| Radial cheilostomatal divisions^a^ | 0 | 0 | 0 | 1 | 1 | 0 | 0 | 1 | 1 | 0 | 0 | 1 |
| Stomatal dimorphism | 0 | 0 | 0 | 1 | 1 | 0 | 0 | 0 | 0 | 0 | 0 | 0 |

^a^See Fürst von Lieven and Sudhaus (2000).

^b^See Fürst von Lieven (2000).

^c^See Kanzaki et al. (2012).

^d^See Herrmann et al. (2013).

^e^See Ragsdale et al. (2014).

*Original in this study.

**Figure 4-source data 1, continued. Matrix of structures tabulated to measure stomatal complexity.** Structures were recorded as described in text. Presence/absence of dimorphism is also given. 0 = absence, 1 = presence.

|  | *Fictor* *stercorarius* | *Fictor* sp. 1 | *Fictor* sp. 2 | *Fuchsnema halleri* | *Fuchsnema* sp. | *Heterorhabditis bacteriophora* | *Koerneria luziae* | *Koerneria* sp. | *Leptojacobus dorci* | *Levipalatum texanum* | *Mehdinema alii* |
| --- | --- | --- | --- | --- | --- | --- | --- | --- | --- | --- | --- |
| **Complexity index** | **5** | **9** | **9** | **1** | **1** | **0** | **4** | **4** | **3** | **3** | **1** |
| Glottoid apparatus^a^ | 0 | 0 | 0 | 0 | 0 | 0 | 0 | 0 | 0 | 0 | 0 |
| Moveable dorsal tooth^a^ | 1 | 1 | 1 | 1 | 1 | 0 | 1 | 1 | 1 | 1 | 1 |
| Fixed dorsal tooth^b^ | 0 | 0 | 0 | 0 | 0 | 0 | 0 | 0 | 0 | 0 | 0 |
| Serratae on anterior gymnostom* | 0 | 1 | 1 | 0 | 0 | 0 | 0 | 0 | 0 | 0 | 0 |
| Pro-/mesostegomatal serratae* | 0 | 1 | 1 | 0 | 0 | 0 | 0 | 0 | 0 | 0 | 0 |
| Right subdorsal denticle^a^ | 0 | 0 | 0 | 0 | 0 | 0 | 0 | 0 | 0 | 0 | 0 |
| Right subventral tooth^a^ | 1 | 1 | 1 | 0 | 0 | 0 | 1 | 1 | 0 | 0 | 0 |
| Right subventral ridge of denticles^c^ | 0 | 0 | 0 | 0 | 0 | 0 | 0 | 0 | 0 | 0 | 0 |
| Left subventral denticle or ridge* | 1 | 1 | 1 | 0 | 0 | 0 | 1 | 1 | 1 | 0 | 0 |
| Paired subventral denticles^a^ | 0 | 0 | 0 | 0 | 0 | 0 | 0 | 0 | 0 | 0 | 0 |
| Duplicate left subventral ridge* | 0 | 1 | 1 | 0 | 0 | 0 | 0 | 0 | 0 | 0 | 0 |
| Right ventral denticle^c^ | 0 | 0 | 0 | 0 | 0 | 0 | 0 | 0 | 0 | 0 | 0 |
| Lateral denticles^a^ | 0 | 0 | 0 | 0 | 0 | 0 | 0 | 0 | 0 | 0 | 0 |
| Pairs of metastegostomatal rods^d^ | 0 | 0 | 0 | 0 | 0 | 0 | 0 | 0 | 0 | 0 | 0 |
| Subventral stegostomatal warts^a^ | 0 | 0 | 0 | 0 | 0 | 0 | 0 | 0 | 0 | 0 | 0 |
| Telostegostomatal ridge^e^ | 0 | 0 | 0 | 0 | 0 | 0 | 0 | 0 | 0 | 1 | 0 |
| Triadial telostegostomatal points^d^ | 1 | 1 | 1 | 0 | 0 | 0 | 0 | 0 | 0 | 0 | 0 |
| Dorsal, basal telostegostomatal fold* | 0 | 0 | 0 | 0 | 0 | 0 | 0 | 0 | 0 | 0 | 0 |
| Articulated apodemes^a^ | 0 | 0 | 0 | 0 | 0 | 0 | 1 | 1 | 0 | 0 | 0 |
| Perradial telostegostomatal knobs^a^ | 0 | 0 | 0 | 0 | 0 | 0 | 0 | 0 | 0 | 0 | 0 |
| Prostegostomatal row of denticles^b^ | 0 | 0 | 0 | 0 | 0 | 0 | 0 | 0 | 0 | 0 | 0 |
| Gymnostomatal denticles^b^ | 0 | 0 | 0 | 0 | 0 | 0 | 0 | 0 | 0 | 0 | 0 |
| Prostego-/gymnostomatal fold^e^ | 0 | 0 | 0 | 0 | 0 | 0 | 0 | 0 | 0 | 1 | 0 |
| Radial gymnostomatal divisions* | 0 | 1 | 1 | 0 | 0 | 0 | 0 | 0 | 0 | 0 | 0 |
| Radial cheilostomatal divisions^a^ | 1 | 1 | 1 | 0 | 0 | 0 | 0 | 0 | 1 | 0 | 0 |
| Stomatal dimorphism | 1 | 1 | 1 | 0 | 0 | 0 | 1 | 1 | 0 | 0 | 0 |

^a^See Fürst von Lieven and Sudhaus (2000).

^b^See Fürst von Lieven (2000).

^c^See Kanzaki et al. (2012).

^d^See Herrmann et al. (2013).

^e^See Ragsdale et al. (2014).

*Original in this study.

**Figure 4-source data 1, continued. Matrix of structures tabulated to measure stomatal complexity.** Structures were recorded as described in text. Presence/absence of dimorphism is also given. 0 = absence, 1 = presence.

|  | *Mesorhabditis acidophila* | *Micoletzkya buetschlii* | *Micoletzkya inedia* | *Micoletzkya* *japonica* | *Micoletzkya* sp. | *Mononchoides* sp. 1 | *Mononchoides* sp. 2 | *Mononchoides* sp. 3 | *Myolaimus byersi* | *Neodiplogaster* *crenatae* | *Neodiplogaster* sp. |
| --- | --- | --- | --- | --- | --- | --- | --- | --- | --- | --- | --- |
| **Complexity index** | **1** | **4** | **4** | **4** | **4** | **8** | **6** | **6** | **0** | **4** | **4** |
| Glottoid apparatus^a^ | 1 | 0 | 0 | 0 | 0 | 0 | 0 | 0 | 0 | 0 | 0 |
| Moveable dorsal tooth^a^ | 0 | 1 | 1 | 1 | 1 | 1 | 1 | 1 | 0 | 1 | 1 |
| Fixed dorsal tooth^b^ | 0 | 0 | 0 | 0 | 0 | 0 | 0 | 0 | 0 | 0 | 0 |
| Serratae on anterior gymnostom* | 0 | 0 | 0 | 0 | 0 | 1 | 0 | 0 | 0 | 0 | 0 |
| Pro-/mesostegomatal serratae* | 0 | 0 | 0 | 0 | 0 | 1 | 0 | 0 | 0 | 0 | 0 |
| Right subdorsal denticle^a^ | 0 | 0 | 0 | 0 | 0 | 1 | 1 | 1 | 0 | 0 | 0 |
| Right subventral tooth^a^ | 0 | 1 | 1 | 1 | 1 | 1 | 1 | 1 | 0 | 1 | 1 |
| Right subventral ridge of denticles^c^ | 0 | 0 | 0 | 0 | 0 | 0 | 0 | 0 | 0 | 0 | 0 |
| Left subventral denticle or ridge* | 0 | 1 | 1 | 1 | 1 | 1 | 1 | 1 | 0 | 0 | 0 |
| Paired subventral denticles^a^ | 0 | 0 | 0 | 0 | 0 | 0 | 0 | 0 | 0 | 0 | 0 |
| Duplicate left subventral ridge* | 0 | 0 | 0 | 0 | 0 | 0 | 0 | 0 | 0 | 0 | 0 |
| Right ventral denticle^c^ | 0 | 0 | 0 | 0 | 0 | 0 | 0 | 0 | 0 | 0 | 0 |
| Lateral denticles^a^ | 0 | 0 | 0 | 0 | 0 | 0 | 0 | 0 | 0 | 0 | 0 |
| Pairs of metastegostomatal rods^d^ | 0 | 0 | 0 | 0 | 0 | 0 | 0 | 0 | 0 | 0 | 0 |
| Subventral stegostomatal warts^a^ | 0 | 0 | 0 | 0 | 0 | 0 | 0 | 0 | 0 | 0 | 0 |
| Telostegostomatal ridge^e^ | 0 | 0 | 0 | 0 | 0 | 0 | 0 | 0 | 0 | 0 | 0 |
| Triadial telostegostomatal points^d^ | 0 | 0 | 0 | 0 | 0 | 0 | 0 | 0 | 0 | 0 | 0 |
| Dorsal, basal telostegostomatal fold* | 0 | 0 | 0 | 0 | 0 | 1 | 1 | 1 | 0 | 0 | 0 |
| Articulated apodemes^a^ | 0 | 0 | 0 | 0 | 0 | 0 | 0 | 0 | 0 | 0 | 0 |
| Perradial telostegostomatal knobs^a^ | 0 | 0 | 0 | 0 | 0 | 0 | 0 | 0 | 0 | 1 | 1 |
| Prostegostomatal row of denticles^b^ | 0 | 0 | 0 | 0 | 0 | 0 | 0 | 0 | 0 | 0 | 0 |
| Gymnostomatal denticles^b^ | 0 | 0 | 0 | 0 | 0 | 0 | 0 | 0 | 0 | 0 | 0 |
| Prostego-/gymnostomatal fold^e^ | 0 | 0 | 0 | 0 | 0 | 0 | 0 | 0 | 0 | 0 | 0 |
| Radial gymnostomatal divisions* | 0 | 0 | 0 | 0 | 0 | 0 | 0 | 0 | 0 | 0 | 0 |
| Radial cheilostomatal divisions^a^ | 0 | 1 | 1 | 1 | 1 | 1 | 1 | 1 | 0 | 1 | 1 |
| Stomatal dimorphism | 0 | 1 | 1 | 1 | 0 | 1 | 1 | 1 | 0 | 0 | 1 |

^a^See Fürst von Lieven and Sudhaus (2000).

^b^See Fürst von Lieven (2000).

^c^See Kanzaki et al. (2012).

^d^See Herrmann et al. (2013).

^e^See Ragsdale et al. (2014).

*Original in this study.

**Figure 4-source data 1, continued. Matrix of structures tabulated to measure stomatal complexity.** Structures were recorded as described in text. Presence/absence of dimorphism is also given. 0 = absence, 1 = presence.

|  | *Odontopharynx longicaudata* | *Oigolaimella attenuata* | *Oigolaimella* sp. | *Oscheius carolinensis* | *Oscheius guentheri* | *Oscheius myriophila* | *Oscheius tipulae* | *Panagrellus redivivus* | *Parapristionchus giblindavisi* | *Parasitodiplogaster maxinema* | *Parasitorhabditis obtusa* |
| --- | --- | --- | --- | --- | --- | --- | --- | --- | --- | --- | --- |
| **Complexity index** | **3** | **3** | **3** | **1** | **1** | **1** | **1** | **0** | **4** | **3** | **0** |
| Glottoid apparatus^a^ | 0 | 0 | 0 | 1 | 1 | 1 | 1 | 0 | 0 | 0 | 0 |
| Moveable dorsal tooth^a^ | 0 | 1 | 1 | 0 | 0 | 0 | 0 | 0 | 1 | 1 | 0 |
| Fixed dorsal tooth^b^ | 1 | 0 | 0 | 0 | 0 | 0 | 0 | 0 | 0 | 0 | 0 |
| Serratae on anterior gymnostom* | 0 | 0 | 0 | 0 | 0 | 0 | 0 | 0 | 0 | 0 | 0 |
| Pro-/mesostegomatal serratae* | 0 | 0 | 0 | 0 | 0 | 0 | 0 | 0 | 0 | 0 | 0 |
| Right subdorsal denticle^a^ | 0 | 0 | 0 | 0 | 0 | 0 | 0 | 0 | 0 | 0 | 0 |
| Right subventral tooth^a^ | 0 | 1 | 1 | 0 | 0 | 0 | 0 | 0 | 1 | 1 | 0 |
| Right subventral ridge of denticles^c^ | 0 | 0 | 0 | 0 | 0 | 0 | 0 | 0 | 0 | 0 | 0 |
| Left subventral denticle or ridge* | 0 | 0 | 0 | 0 | 0 | 0 | 0 | 0 | 1 | 0 | 0 |
| Paired subventral denticles^a^ | 0 | 0 | 0 | 0 | 0 | 0 | 0 | 0 | 0 | 0 | 0 |
| Duplicate left subventral ridge* | 0 | 0 | 0 | 0 | 0 | 0 | 0 | 0 | 0 | 0 | 0 |
| Right ventral denticle^c^ | 0 | 0 | 0 | 0 | 0 | 0 | 0 | 0 | 0 | 0 | 0 |
| Lateral denticles^a^ | 0 | 0 | 0 | 0 | 0 | 0 | 0 | 0 | 0 | 0 | 0 |
| Pairs of metastegostomatal rods^d^ | 0 | 0 | 0 | 0 | 0 | 0 | 0 | 0 | 0 | 0 | 0 |
| Subventral stegostomatal warts^a^ | 0 | 0 | 0 | 0 | 0 | 0 | 0 | 0 | 0 | 0 | 0 |
| Telostegostomatal ridge^e^ | 0 | 0 | 0 | 0 | 0 | 0 | 0 | 0 | 0 | 0 | 0 |
| Triadial telostegostomatal points^d^ | 0 | 0 | 0 | 0 | 0 | 0 | 0 | 0 | 0 | 0 | 0 |
| Dorsal, basal telostegostomatal fold* | 0 | 0 | 0 | 0 | 0 | 0 | 0 | 0 | 0 | 0 | 0 |
| Articulated apodemes^a^ | 0 | 0 | 0 | 0 | 0 | 0 | 0 | 0 | 0 | 1 | 0 |
| Perradial telostegostomatal knobs^a^ | 0 | 0 | 0 | 0 | 0 | 0 | 0 | 0 | 0 | 0 | 0 |
| Prostegostomatal row of denticles^b^ | 1 | 0 | 0 | 0 | 0 | 0 | 0 | 0 | 0 | 0 | 0 |
| Gymnostomatal denticles^b^ | 1 | 0 | 0 | 0 | 0 | 0 | 0 | 0 | 0 | 0 | 0 |
| Prostego-/gymnostomatal fold^e^ | 0 | 0 | 0 | 0 | 0 | 0 | 0 | 0 | 0 | 0 | 0 |
| Radial gymnostomatal divisions* | 0 | 0 | 0 | 0 | 0 | 0 | 0 | 0 | 0 | 0 | 0 |
| Radial cheilostomatal divisions^a^ | 0 | 1 | 1 | 0 | 0 | 0 | 0 | 0 | 1 | 0 | 0 |
| Stomatal dimorphism | 0 | 0 | 0 | 0 | 0 | 0 | 0 | 0 | 1 | 1 | 0 |

^a^See Fürst von Lieven and Sudhaus (2000).

^b^See Fürst von Lieven (2000).

^c^See Kanzaki et al. (2012).

^d^See Herrmann et al. (2013).

^e^See Ragsdale et al. (2014).

*Original in this study.

**Figure 4-source data 1, continued. Matrix of structures tabulated to measure stomatal complexity.** Structures were recorded as described in text. Presence/absence of dimorphism is also given. 0 = absence, 1 = presence.

|  | *Paroigolaimella micrura* | *Paroigolaimella stresemanni* | *Pellioditis* sp. | *Pelodera cylindrica* | *Pelodera strongyloides* | *Pelodera teres* | *Poikilolaimus floridensis* | *Poikilolaimus oxycercus* | *Pristionchus elegans* | *Pristionchus fissidentatus* | *Pristionchus maupasi* |
| --- | --- | --- | --- | --- | --- | --- | --- | --- | --- | --- | --- |
| **Complexity index** | **3** | **3** | **1** | **1** | **1** | **1** | **1** | **1** | **5** | **6** | **4** |
| Glottoid apparatus^a^ | 0 | 0 | 1 | 1 | 1 | 1 | 1 | 1 | 0 | 0 | 0 |
| Moveable dorsal tooth^a^ | 1 | 1 | 0 | 0 | 0 | 0 | 0 | 0 | 1 | 1 | 1 |
| Fixed dorsal tooth^b^ | 0 | 0 | 0 | 0 | 0 | 0 | 0 | 0 | 0 | 0 | 0 |
| Serratae on anterior gymnostom* | 0 | 0 | 0 | 0 | 0 | 0 | 0 | 0 | 1 | 0 | 0 |
| Pro-/mesostegomatal serratae* | 0 | 0 | 0 | 0 | 0 | 0 | 0 | 0 | 0 | 0 | 0 |
| Right subdorsal denticle^a^ | 0 | 0 | 0 | 0 | 0 | 0 | 0 | 0 | 0 | 0 | 0 |
| Right subventral tooth^a^ | 0 | 0 | 0 | 0 | 0 | 0 | 0 | 0 | 1 | 1 | 1 |
| Right subventral ridge of denticles^c^ | 0 | 0 | 0 | 0 | 0 | 0 | 0 | 0 | 0 | 1 | 0 |
| Left subventral denticle or ridge* | 0 | 0 | 0 | 0 | 0 | 0 | 0 | 0 | 1 | 1 | 1 |
| Paired subventral denticles^a^ | 0 | 0 | 0 | 0 | 0 | 0 | 0 | 0 | 0 | 0 | 0 |
| Duplicate left subventral ridge* | 0 | 0 | 0 | 0 | 0 | 0 | 0 | 0 | 0 | 0 | 0 |
| Right ventral denticle^c^ | 0 | 0 | 0 | 0 | 0 | 0 | 0 | 0 | 0 | 1 | 0 |
| Lateral denticles^a^ | 0 | 0 | 0 | 0 | 0 | 0 | 0 | 0 | 0 | 0 | 0 |
| Pairs of metastegostomatal rods^d^ | 0 | 0 | 0 | 0 | 0 | 0 | 0 | 0 | 0 | 0 | 0 |
| Subventral stegostomatal warts^a^ | 1 | 1 | 0 | 0 | 0 | 0 | 0 | 0 | 0 | 0 | 0 |
| Telostegostomatal ridge^e^ | 0 | 0 | 0 | 0 | 0 | 0 | 0 | 0 | 0 | 0 | 0 |
| Triadial telostegostomatal points^d^ | 0 | 0 | 0 | 0 | 0 | 0 | 0 | 0 | 0 | 0 | 0 |
| Dorsal, basal telostegostomatal fold* | 0 | 0 | 0 | 0 | 0 | 0 | 0 | 0 | 0 | 0 | 0 |
| Articulated apodemes^a^ | 0 | 0 | 0 | 0 | 0 | 0 | 0 | 0 | 0 | 0 | 0 |
| Perradial telostegostomatal knobs^a^ | 0 | 0 | 0 | 0 | 0 | 0 | 0 | 0 | 0 | 0 | 0 |
| Prostegostomatal row of denticles^b^ | 0 | 0 | 0 | 0 | 0 | 0 | 0 | 0 | 0 | 0 | 0 |
| Gymnostomatal denticles^b^ | 0 | 0 | 0 | 0 | 0 | 0 | 0 | 0 | 0 | 0 | 0 |
| Prostego-/gymnostomatal fold^e^ | 0 | 0 | 0 | 0 | 0 | 0 | 0 | 0 | 0 | 0 | 0 |
| Radial gymnostomatal divisions* | 0 | 0 | 0 | 0 | 0 | 0 | 0 | 0 | 0 | 0 | 0 |
| Radial cheilostomatal divisions^a^ | 1 | 1 | 0 | 0 | 0 | 0 | 0 | 0 | 1 | 1 | 1 |
| Stomatal dimorphism | 0 | 0 | 0 | 0 | 0 | 0 | 0 | 0 | 0 | 1 | 1 |

^a^See Fürst von Lieven and Sudhaus (2000).

^b^See Fürst von Lieven (2000).

^c^See Kanzaki et al. (2012).

^d^See Herrmann et al. (2013).

^e^See Ragsdale et al. (2014).

*Original in this study.**Figure 4-source data 1, continued. Matrix of structures tabulated to measure stomatal complexity.** Structures were recorded as described in text. Presence/absence of dimorphism is also given. 0 = absence, 1 = presence.

|  | *Pristionchus pacificus* | *Pristionchus uniformis* | *Protorhabditis* sp. | *Pseudodiplogasteroides* sp. | *Rhabditidoides* sp. | *Rhabditis brassicae* | *Rhabditis colombiana* | *Rhabditis rainai* | *Rhabditoides inermis* | *Rhabditolaimus* sp. 1 | *Rhabditolaimus* sp. 2 |
| --- | --- | --- | --- | --- | --- | --- | --- | --- | --- | --- | --- |
| **Complexity index** | **4** | **4** | **0** | **2** | **1** | **1** | **1** | **1** | **1** | **0** | **0** |
| Glottoid apparatus^a^ | 0 | 0 | 0 | 0 | 0 | 1 | 1 | 1 | 1 | 0 | 0 |
| Moveable dorsal tooth^a^ | 1 | 1 | 0 | 1 | 1 | 0 | 0 | 0 | 0 | 0 | 0 |
| Fixed dorsal tooth^b^ | 0 | 0 | 0 | 0 | 0 | 0 | 0 | 0 | 0 | 0 | 0 |
| Serratae on anterior gymnostom* | 0 | 0 | 0 | 0 | 0 | 0 | 0 | 0 | 0 | 0 | 0 |
| Pro-/mesostegomatal serratae* | 0 | 0 | 0 | 0 | 0 | 0 | 0 | 0 | 0 | 0 | 0 |
| Right subdorsal denticle^a^ | 0 | 0 | 0 | 0 | 0 | 0 | 0 | 0 | 0 | 0 | 0 |
| Right subventral tooth^a^ | 1 | 1 | 0 | 0 | 0 | 0 | 0 | 0 | 0 | 0 | 0 |
| Right subventral ridge of denticles^c^ | 0 | 0 | 0 | 0 | 0 | 0 | 0 | 0 | 0 | 0 | 0 |
| Left subventral denticle or ridge* | 1 | 1 | 0 | 0 | 0 | 0 | 0 | 0 | 0 | 0 | 0 |
| Paired subventral denticles^a^ | 0 | 0 | 0 | 0 | 0 | 0 | 0 | 0 | 0 | 0 | 0 |
| Duplicate left subventral ridge* | 0 | 0 | 0 | 0 | 0 | 0 | 0 | 0 | 0 | 0 | 0 |
| Right ventral denticle^c^ | 0 | 0 | 0 | 0 | 0 | 0 | 0 | 0 | 0 | 0 | 0 |
| Lateral denticles^a^ | 0 | 0 | 0 | 0 | 0 | 0 | 0 | 0 | 0 | 0 | 0 |
| Pairs of metastegostomatal rods^d^ | 0 | 0 | 0 | 0 | 0 | 0 | 0 | 0 | 0 | 0 | 0 |
| Subventral stegostomatal warts^a^ | 0 | 0 | 0 | 0 | 0 | 0 | 0 | 0 | 0 | 0 | 0 |
| Telostegostomatal ridge^e^ | 0 | 0 | 0 | 0 | 0 | 0 | 0 | 0 | 0 | 0 | 0 |
| Triadial telostegostomatal points^d^ | 0 | 0 | 0 | 0 | 0 | 0 | 0 | 0 | 0 | 0 | 0 |
| Dorsal, basal telostegostomatal fold* | 0 | 0 | 0 | 0 | 0 | 0 | 0 | 0 | 0 | 0 | 0 |
| Articulated apodemes^a^ | 0 | 0 | 0 | 0 | 0 | 0 | 0 | 0 | 0 | 0 | 0 |
| Perradial telostegostomatal knobs^a^ | 0 | 0 | 0 | 0 | 0 | 0 | 0 | 0 | 0 | 0 | 0 |
| Prostegostomatal row of denticles^b^ | 0 | 0 | 0 | 0 | 0 | 0 | 0 | 0 | 0 | 0 | 0 |
| Gymnostomatal denticles^b^ | 0 | 0 | 0 | 0 | 0 | 0 | 0 | 0 | 0 | 0 | 0 |
| Prostego-/gymnostomatal fold^e^ | 0 | 0 | 0 | 1 | 0 | 0 | 0 | 0 | 0 | 0 | 0 |
| Radial gymnostomatal divisions* | 0 | 0 | 0 | 0 | 0 | 0 | 0 | 0 | 0 | 0 | 0 |
| Radial cheilostomatal divisions^a^ | 1 | 1 | 0 | 0 | 0 | 0 | 0 | 0 | 0 | 0 | 0 |
| Stomatal dimorphism | 0 | 1 | 0 | 0 | 0 | 0 | 0 | 0 | 0 | 0 | 0 |

^a^See Fürst von Lieven and Sudhaus (2000).

^b^See Fürst von Lieven (2000).

^c^See Kanzaki et al. (2012).

^d^See Herrmann et al. (2013).

^e^See Ragsdale et al. (2014).

*Original in this study.**Figure 4-source data 1, continued. Matrix of structures tabulated to measure stomatal complexity.** Structures were recorded as described in text. Presence/absence of dimorphism is also given. 0 = absence, 1 = presence.

|  | *Sachsia zurstrasseni* | *Strongyloides ratti* | *Sudhausia aristotokia* | *Sudhausia crassa* | *Teratodiplogaster* sp. 1 | *Teratodiplogaster* sp. 2 | *Teratorhabditis mariannae* | *Teratorhabditis palmarum* | *Teratorhabditis synpapillata* | *Tylopharynx foetida* | *Zeldia punctata* |
| --- | --- | --- | --- | --- | --- | --- | --- | --- | --- | --- | --- |
| **Complexity index** | **2** | **0** | **3** | **3** | **2** | **2** | **1** | **1** | **1** | **3** | **0** |
| Glottoid apparatus^a^ | 0 | 0 | 0 | 0 | 0 | 0 | 1 | 1 | 1 | 0 | 0 |
| Moveable dorsal tooth^a^ | 1 | 0 | 1 | 1 | 1 | 1 | 0 | 0 | 0 | 1 | 0 |
| Fixed dorsal tooth^b^ | 0 | 0 | 0 | 0 | 0 | 0 | 0 | 0 | 0 | 0 | 0 |
| Serratae on anterior gymnostom* | 0 | 0 | 0 | 0 | 0 | 0 | 0 | 0 | 0 | 0 | 0 |
| Pro-/mesostegomatal serratae* | 0 | 0 | 0 | 0 | 0 | 0 | 0 | 0 | 0 | 0 | 0 |
| Right subdorsal denticle^a^ | 0 | 0 | 0 | 0 | 0 | 0 | 0 | 0 | 0 | 0 | 0 |
| Right subventral tooth^a^ | 0 | 0 | 0 | 0 | 1 | 1 | 0 | 0 | 0 | 1 | 0 |
| Right subventral ridge of denticles^c^ | 0 | 0 | 0 | 0 | 0 | 0 | 0 | 0 | 0 | 0 | 0 |
| Left subventral denticle or ridge* | 0 | 0 | 0 | 0 | 0 | 0 | 0 | 0 | 0 | 0 | 0 |
| Paired subventral denticles^a^ | 0 | 0 | 0 | 0 | 0 | 0 | 0 | 0 | 0 | 0 | 0 |
| Duplicate left subventral ridge* | 0 | 0 | 0 | 0 | 0 | 0 | 0 | 0 | 0 | 0 | 0 |
| Right ventral denticle^c^ | 0 | 0 | 0 | 0 | 0 | 0 | 0 | 0 | 0 | 0 | 0 |
| Lateral denticles^a^ | 0 | 0 | 0 | 0 | 0 | 0 | 0 | 0 | 0 | 0 | 0 |
| Pairs of metastegostomatal rods^d^ | 0 | 0 | 1 | 1 | 0 | 0 | 0 | 0 | 0 | 0 | 0 |
| Subventral stegostomatal warts^a^ | 0 | 0 | 0 | 0 | 0 | 0 | 0 | 0 | 0 | 0 | 0 |
| Telostegostomatal ridge^e^ | 0 | 0 | 0 | 0 | 0 | 0 | 0 | 0 | 0 | 0 | 0 |
| Triadial telostegostomatal points^d^ | 0 | 0 | 1 | 1 | 0 | 0 | 0 | 0 | 0 | 0 | 0 |
| Dorsal, basal telostegostomatal fold* | 0 | 0 | 0 | 0 | 0 | 0 | 0 | 0 | 0 | 0 | 0 |
| Articulated apodemes^a^ | 0 | 0 | 0 | 0 | 0 | 0 | 0 | 0 | 0 | 0 | 0 |
| Perradial telostegostomatal knobs^a^ | 0 | 0 | 0 | 0 | 0 | 0 | 0 | 0 | 0 | 1 | 0 |
| Prostegostomatal row of denticles^b^ | 0 | 0 | 0 | 0 | 0 | 0 | 0 | 0 | 0 | 0 | 0 |
| Gymnostomatal denticles^b^ | 0 | 0 | 0 | 0 | 0 | 0 | 0 | 0 | 0 | 0 | 0 |
| Prostego-/gymnostomatal fold^e^ | 0 | 0 | 0 | 0 | 0 | 0 | 0 | 0 | 0 | 0 | 0 |
| Radial gymnostomatal divisions* | 0 | 0 | 0 | 0 | 0 | 0 | 0 | 0 | 0 | 0 | 0 |
| Radial cheilostomatal divisions^a^ | 1 | 0 | 0 | 0 | 0 | 0 | 0 | 0 | 0 | 0 | 0 |
| Stomatal dimorphism | 0 | 0 | 0 | 0 | 0 | 0 | 0 | 0 | 0 | 0 | 0 |

^a^See Fürst von Lieven and Sudhaus (2000).

^b^See Fürst von Lieven (2000).

^c^See Kanzaki et al. (2012).

^d^See Herrmann et al. (2013).

^e^See Ragsdale et al. (2014).

*Original in this study.
